# The Effect of Denture Cleansing Solutions on the Retention of Precision Attachments: An In Vitro Study

**DOI:** 10.3390/ijerph19074345

**Published:** 2022-04-05

**Authors:** Gonca Deste Gokay, Serhat Emre Ozkir, Thomas Gerhard Wolf, Gulsum Gokcimen, Nergiz Rona, Mehmet Bicer, Burak Yilmaz

**Affiliations:** 1 Department of Prosthodontics, Faculty of Dentistry, Bursa Uludag University, Bursa 16059, Turkey; goncadeste@uludag.edu.tr; 2 Private Dental Clinic, Eskişehir 26000, Turkey; emreozkir@yahoo.com (S.E.O.); mehmetbicer2656@gmail.com (M.B.); 3 Department of Restorative, Preventive and Pediatric Dentistry, School of Dental Medicine, University of Bern, CH-3010 Bern, Switzerland; thomas.wolf@zmk.unibe.ch; 4 Department of Periodontology and Operative Dentistry, University Medical Center, Johannes Gutenberg-University Mainz, D-553131 Mainz, Germany; 5 Department of Prosthodontics, Faculty of Dentistry, Afyonkarahisar Health Sciences University, Afyonkarahisar 03030, Turkey; gulsum.gokcimen@afsu.edu.tr (G.G.); nergiz.rona@afsu.edu.tr (N.R.); 6 Department of Reconstructive Dentistry and Gerodontology, School of Dental Medicine, University of Bern, CH-3010 Bern, Switzerland; 7 Department of Restorative and Prosthetic Dentistry, The Ohio State University, Columbus, OH 43210, USA

**Keywords:** precision attachments, cleansing solutions, partial removable dentures, tap water, denture retention

## Abstract

This study aimed to investigate the effect of different cleansing solutions on the retention of precision attachments. A precision attachment patrix was embedded into acrylic resin and the matrix was placed onto the patrix. The red (high retention, 8 N), yellow (regular retention, 6 N), and green (reduced retention, 4 N) plastic matrixes of the attachments (*n* = 32) were soaked in three different denture cleansing solutions (sodium laureth sulfate, sodium bicarbonate-sodium perborate, sodium bicarbonate) for a duration simulating 6 months of clinical use. The control group was soaked in tap water. A universal testing machine was used to measure the retention values of attachments after they were soaked in denture cleansers. The retention values were compared among the groups with repeated-measures analysis of variance followed by the Tukey HSD test (*p* = 0.05). Yellow attachments were affected by sodium laureth sulfate, sodium bicarbonate-sodium perborate, and water (*p* = 0.012). Green attachments’ retention increased after immersion in sodium laureth sulfate (*p* = 0.04) and water (*p* = 0.02). Red attachments’ retention increased after immersion in sodium laureth sulfate or sodium bicarbonate-sodium perborate (*p* = 0.045). Water did not affect the retention of red attachments. Because sodium bicarbonate tablets did not affect the retention of attachments, clinicians may recommend their use as a cleanser. Clinicians also may inform patients using fixed and removable partial prostheses with precision attachments of a possible increase in retention after the use of sodium laureth sulfate or when using sodium bicarbonate-sodium perborate with yellow and red attachments.

## 1. Introduction

Even though implant-supported fixed restorations and fixed partial dentures (FPD) have advantages, they are not always feasible due to patient-related contraindications or financial limitations [1]. A combination of fixed and removable partial dentures (RPD) with precision attachments are still used to restore function and esthetics for patients with missing teeth [2]. With this option, time consuming, invasive, and costly surgical procedures may be eliminated [3,4,5]. A precision attachment is used instead of clasps for fixation, retention, and stabilization of a dental prosthesis [6]. Ball and bolt attachments are economical and commonly used. Intermaxillary space, load distribution on the mucosa and teeth, and degree of desired retention affect the decision when selecting the attachments [1,4]. The retention characteristics of attachments change by type, design, and wear. The wear mechanisms are tribochemical reaction, abrasion, adhesion, and surface disruption [7,8,9,10]. It has been reported that RPDs which are retained by precision attachments have a role in improving the quality of life in partially dentate patients [11].

Following the delivery of removable dentures with attachments, cleaning procedures become crucial as the matrixes of the dentures are difficult to clean. In order to clean the dentures, mechanical, chemical procedures, or a combination of both can be used [12,13]. Most of denture wearers use mechanical procedures (brushing) to clean their dentures [14]. Even though brushing is inexpensive and mostly easy, brushing technique is crucial for long term adequate care of the dentures, and abrasive properties of dentifrices may damage the denture [15,16]. Different chemical cleansing solutions are commercially available and household products are also used [13]. Patients immerse their dentures in sodium hypochlorite (NaOCl), effervescent tablets containing sodium bicarbonate, sodium perborate etc. [2,17]. NaOCl is affordable, easy to use, remove stains, dissolves organic substances, and it is bactericidal. However, corrosion potential and bleaching effects are the main disadvantages of this solution [2,18]. 

Previous studies on RPDs have focused on the effects of cleansing agents on the acrylic resin [2,12,13]. Some studies have focused on the effect of cleansing solutions on the attachments: however, the attachments tested were manufactured for implant-retained dentures [6,7,16]. To the authors’ knowledge, no study has investigated the effect of cleansing solutions on the precision attachments of tooth-retained RPDs.

A denture is removed and inserted four times on a daily basis [16]. The removal and replacement cycles may wear the attachments, and the denture cleansers may have corrosive effects [19]. Accordingly, it may be beneficial to compare retentive properties of attachments post-insertion, and not limit their assessments to the initial stage only [20]. 

Preci-Vertix is a bolt-type semi-precision extracoronal attachment for RPDs. It has advantages such as simple fabrication procedures and servicing, improved esthetics, cost-effectiveness and excellent patient comfort. Matrixes of a precision attachment system are fabricated from various polymers and available in three color-coded retention values (frictional features): yellow for standard, white/green for decreased, and red for increased retention [21].

Previous studies have compared the effects of cleansing agents on stud, bar-clip, and magnet attachments, which are commonly used to retain mandibular overdentures [7]. Even though the precision attachments for RPDs have parts similar to those used for overdentures, to the authors’ knowledge, the effect of cleansing agents on their retention has not been studied. The aim of this study was to evaluate the effects of various cleansing solutions on the retention of precision attachments with different retention. The first null hypothesis of this study was that the retention of attachments would not be affected by the cleansing solution. The second null hypothesis was that the retention change after immersion would not be different for attachments with different retention.

## 2. Materials and Methods

**Figure 1:**
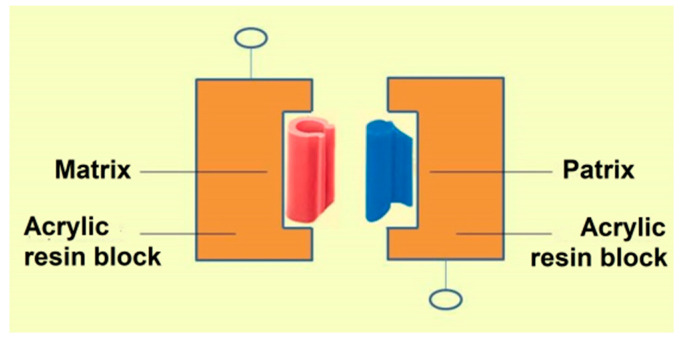
Test arrangement.

No human subjects or animals were involved in this study, and no informed consent was required because the study was performed in vitro on the dental materials. To simulate removable dentures with precision attachments, conventional methods were used and the patrix of the attachments were cast in chromium-cobalt (Cr-Co) alloy. The patrix was placed inside the wax block with a parallelometer. The wax block was placed in a brass flask with Type III dental stone. The flask was placed in a boil-out tank for 5 min, the wax was removed, and the flask was left to cool. Heat-polymerized acrylic resin was prepared and packed in the stone mold and polymerized according to the manufacturer’s instructions. The plastic matrix (female part) of the attachment was placed on the patrix (male part) and the Cr-Co housing was prepared by conventional lost wax technique. An acrylic resin block with a hollow was prepared and the metal housing was placed in the acrylic resin block. The housing was stabilized with an autopolymerizing acrylic resin (Figure 1). 

The denture cleansing solutions used were a sodium bicarbonate-sodium perborate tablet (Protefix Active Cleanser; PAC), a sodium laureth sulfate gel (Aktident Cleansing Gel; AKG), and a sodium bicarbonate tablet (Aktident Cleansing Tablet; AKT). Tap water (TDS = 50–70 mg/L) was used as the control. Eight specimens of red (high retention, 8 N), yellow (regular retention, 6 N), and green (reduced retention, 4 N) matrixes of a precision attachment system (Bredent Medical GmbH & Co. KG, Senden, Germany) were soaked in each cleansing solution [22] (Table 1). The matrixes were soaked without being connected with the patrix during the simulation. The attachments were fully immersed in cleansing solutions. The attachments were immersed in beakers containing 250 mL of each solution according to the manufacturers’ instructions to simulate 6 months of cleansing. The immersion schedule of cleansing solutions is shown in Table 2. The solutions and tap water were refreshed for the next process on a daily basis and the attachments were rinsed under running tap water for 15 s between solution changes. 

The attachments were tested for retention changes in a Universal Testing Machine. A cross-head speed with 50 mm/min was applied on attachments until the matrix separated from the patrix [6,16]. Each attachment was subjected to 12 cycles of insertion and removal, and each cycle involved complete separation from the patrix [20]. At the end of each cycle, the plastic matrix was replaced with a new one. The initial dislodgment values (N) were recorded before immersion in solutions to later calculate the mean maximum dislodgement forces for each group. After simulating six months of clinical use by immersing the attachments in test solutions, the pull-out tests were repeated and maximum dislodgement forces were recorded to calculate the mean maximum dislodgement forces after immersion.

Change in retention (%) after immersion was calculated by the formula [16];

$$\frac{initial\;retention-final\;retention}{initial\;retention}\times 100$$

The changes in retention data after six months of simulated soaking in denture cleansers were compared between the groups by using a repeated measurement ANOVA followed by Tukey HSD Test. *p* ≤ 0.05 was considered significant. 

## 3. Results

**Figure 2:**
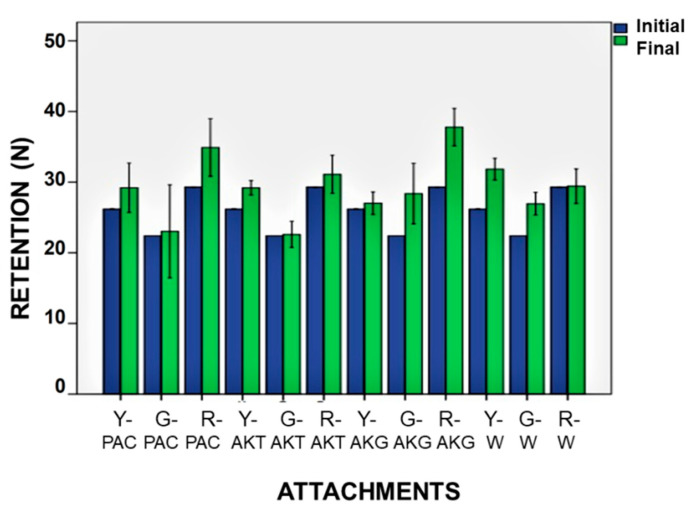
Retention values (N) before and after cleansing (Y: yellow, G: green, R: red, PAC: Protefix Active Cleanser, AKT: Aktident Cleansing Tablet, AKG: Aktident Cleansing Gel, W: water).

According to the Levene test of homogeneity, the variables were normally distributed. According to the ANOVA results, the solution and the attachment type interaction was significant (*p* < 0.05) (Table 3). The retention of some attachments was significantly affected by the cleansing solutions (*p* < 0.05) (Figure 2). For attachments soaked in water, mean (±SD) retention values were 31.84 N (±1.84 N) for yellow, 26.94 N (±1.52 N) for green, and 29.42 N (±1.53 N) for red attachments. For attachments soaked in sodium bicarbonate-sodium perborate (PAC) solution, the mean retention values (±SD) were 29.21 N (±4.17 N) for yellow, 23.03 N (±6.27 N) for green, and 34.91 N (±2.56 N) for red attachments. For attachments soaked in sodium bicarbonate (AKT) solution, the mean retention values were 29.18N (±1.22 N) for yellow, 22.60 N (±1.76 N) for green, and 31.10 N (±1.68 N) for red attachments. For attachments soaked in sodium laureth sulfate (AKG) gel, the retention values were 27.02 N (±1.72 N) for yellow, 28.38 N (±4.08 N) for green and 37.78 N (±1.66 N) for red attachments.

**Figure 3:**
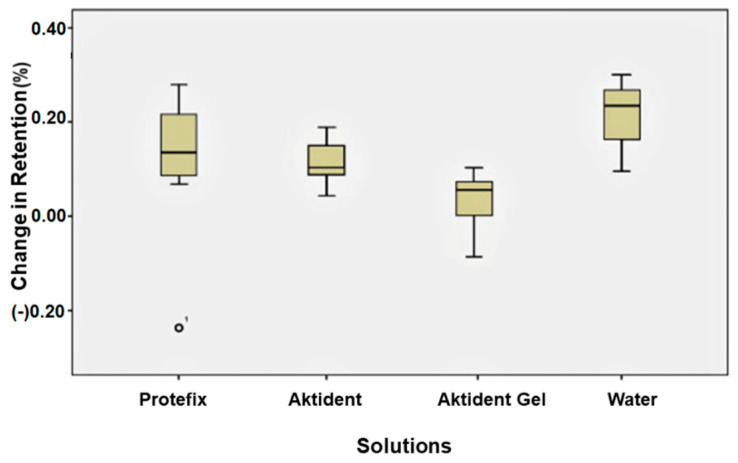
Percent change in retention values for yellow attachments after immersion in different cleansing solutions.

**Figure 4:**
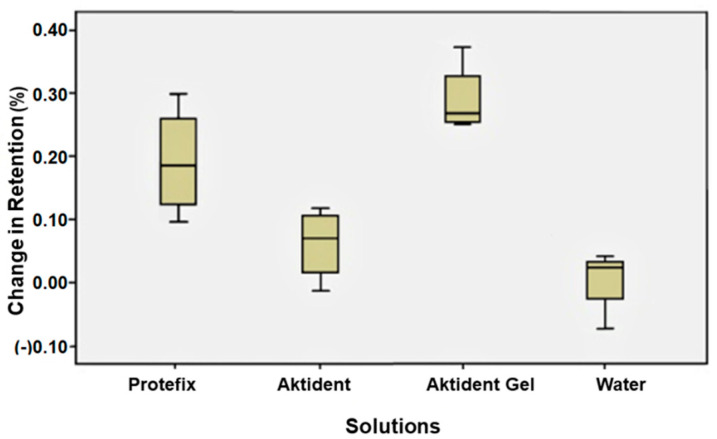
Percent change in retention values for green attachments after immersion in different cleansing solutions.

**Figure 5:**
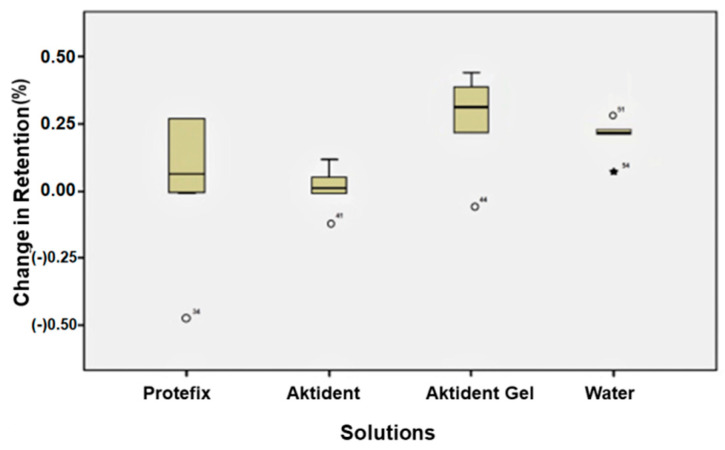
Percent change in retention values for red attachments after immersion in different cleansing solutions.

The retention of yellow attachments increased after immersion in AKG, PAC, or water (*p* = 0.012) (Figure 3). The differences in retention of green attachments after immersion in different solutions were statistically significant (*p* = 0.028); the retention increased after immersion in AKG (*p* = 0.04) or water (*p* = 0.02) (Figure 4). The retention of red attachments increased after immersion in AKG or PAC (*p* = 0.045). Although the retention value of the red attachment increased after soaking in AKT, the change was not statistically significant (*p* > 0.05). Water had no effect on the retention of red attachments (Figure 5). 

## 4. Discussion

The first null hypothesis of this study was rejected as the solutions significantly affected the retention of attachments. The second null hypothesis was also rejected as the amount of change in retention varied amongst attachments depending on the cleansing agent used. Because matrix plastics in different colors provide different retention properties, the composition of the material may differ and the response/reaction of the attachments to different cleansing agents may change. In addition, although some retention changes were statistically insignificant, all retention values increased. The increase in retention values indicates that the attachments possibly harden during the cleansing period. It was unexpected that the retention values of the control group would change as the attachments were soaked in tap water. There should be communication with patients regarding a possible increase in retention after the use of sodium laureth sulfate or when sodium bicarbonate-sodium perborate is used with yellow and red attachments. Because sodium bicarbonate tablets did not affect the retention of attachments, clinicians may recommend their use as a cleanser. 

In a previous study, it was stated that denture cleanser tablets or chlorhexidine mouthwash were compatible for dentures, whereas NaOCl (bleach) or daily-use mouthwashes caused significant damage to the acrylic resin [13]. Commercially-available effervescent cleansing agents generally contain sodium perborate and sodium bicarbonate [12]. During the dissolution in water, alkaline peroxide solution forms, which removes the debris mechanically, and sodium perborate in the structure decomposes. In the present study, sodium bicarbonate containing (AKT) and both sodium bicarbonate and sodium perborate containing (PAC) effervescent tablets and sodium laureth sulfate containing gel (AKG) were used. All these solutions were found to be effective in reducing the biofilm on the dentures [23]. In addition to the effect of solutions on retention values, color changes of the retentive elements were also reported [23]. Kurkçuoglu et al. [20] evaluated the effects of sodium bicarbonate and sodium perborate containing solutions on Locator attachments and found that sodium bicarbonate affected the retention values of soft and rigid attachments, whereas sodium bicarbonate did not affect the medium-retention Locator attachment. Nguyen et al. [6] and You et al. [16] investigated the effects of different cleansing solutions on pink Locator attachment and found lower peak-dislodgement force when sodium perborate solutions were used. All three studies also investigated NaOCl and found detrimental effects on Locator matrixes. In the present study, authors did not use NaOCl as this solution has a high corrosive effect on metals. 

In a study evaluating the effect of simulated function on the retention of the Hader bar-clip overdenture prosthesis, Breeding et al. [24] observed a 30% reduction in retention (maximum dislodgement force) after the initial removal of a single yellow Hader clip from the Hader bar. The reduction in retention reached a plateau by the 12th removal. Twelve cycles of insertion-removal were adopted in the present study in light of the methods used in the above-mentioned study and to be able to make potential comparisons with studies which used a similar protocol. Attachments with different retentive values were affected differently from cleansing solutions in previous studies [6,16,20,25]. Future studies should be conducted to investigate the chemical alterations in attachments and solutions. Because there are many types of attachments and varied manufacturers for RPDs or overdentures, the materials used and their compositions may be affected differently when immersed in solutions. 

With the improvements in dental technology, there are treatment alternatives for partial edentulism such as removable partial dentures manufactured with new-generation materials and implant-supported fixed restorations [4,5,19]. Implant-supported restorations are becoming the dominant treatment procedure for partial or complete edentulism. However, there are many factors influencing the definitive treatment plan such as the financial situation, systemic and local health conditions, and social needs. Kiesow et al. [13] reported in their study that the patient satisfaction from RPDs with precision attachments did not significantly differ from the patient satisfaction obtained with conventional fixed restorations. This being said, RPDs are still one of the options for partially edentulous patients, and any knowledge that may improve the maintenance of removable dentures can be considered beneficial for patients. The application duration of solutions should be extended in future studies to investigate their effects on the retention of RPD attachments in the long-term. The results of this in vitro study should be corroborated with clinical studies and different attachment systems should be also tested. Retention of precision attachments will change and different systems should be investigated to help guide clinicians. In future studies, the effects of cleaning solutions on the characteristics of materials in precision-attachments should be analyzed, which may further clarify the relationship between the material characteristics and retention capabilities. Only the chemical cleaning effects of cleansing agents were considered in the present study and the fact that a brush was not used with the gel cleanser is a limitation. Different results may be obtained if the gel is applied with a brush.

## 5. Conclusions

Within the limitations of this in vitro study, the following conclusions were drawn:

1. Tablets containing sodium bicarbonate-sodium perborate (PAC) and gel containing sodium laureth sulfate (AKG) increased the retention of tested attachments.

2. Water increased the retention of yellow and green attachments.

3. The effect of sodium bicarbonate (AKT) on the retention of attachments was not significant.

## Figures and Tables

**Table 1:** Attachments tested.

Type of the Attachment	Frictional Feature	Number of Specimens	Number of Pulls for Each Attachment
Yellow bolt attachment	Regular friction-6 N	32	12
Green bolt attachment	Reduced friction-4 N	32	12
Red bolt attachment	High retention-8 N	32	12

**Table 2:** Tested denture cleansing solutions and cleaning schedule.

Cleansing Solution	Brand Name and Manufacturer	Time per Day	Total Immersion Time
Sodium bicarbonate-sodium perborate	Protefix Active Cleanser, Queisser Pharma	15 min	540 min (6 months)
Sodium laureth sulfate	Aktident Cleansing Gel, Helago Pharma	2 min	360 min (6 months)
Sodium bicarbonate	Aktident Cleansing Tablet, Helago Pharma	15 min	540 min (6 months)
Tap water		8 h	1440 min (6 months)

**Table 3:** Test of between-subject effects.

Source	Type III Sum of Squares	Df	Mean Square	F	Sig.
Corrected model	0.623	11	0.057	3.637	0.001
Intercept	1.072	1	1.072	68.849	0.000
Attachment	0.003	2	0.002	0.109	0.897
Cleansing agent	0.155	3	0.052	3.313	0.026
Attachment x Agent	0.482	6	0.080	5.160	0.000 ^1^
Error	0.918	59	0.016		
Total	2.684	71			
Corrected total	1.541	70			

^1^ indicates significant difference *p* < 0.05.

## Data Availability

The data are not publicly available due to privacy restrictions.
